# Mendelian randomisation for psychiatry: how does it work, and what can it tell us?

**DOI:** 10.1038/s41380-021-01173-3

**Published:** 2021-06-04

**Authors:** Robyn E. Wootton, Hannah J. Jones, Hannah M. Sallis

**Affiliations:** 1 MRC Integrative Epidemiology Unit, University of Bristol, Bristol, UK; 2 Department of Population Health Sciences, Bristol Medical School, University of Bristol, Bristol, UK; 3 Nic Waals Institute, Lovisenberg Diaconal Hospital, Oslo, Norway; 4 National Institute for Health Research Bristol Biomedical Research Centre, University Hospitals Bristol NHS Foundation Trust, University of Bristol, Bristol, UK; 5 Centre for Academic Mental Health, Population Health Sciences, Bristol Medical School, University of Bristol, Bristol, UK; 6 School of Psychological Science, University of Bristol, Bristol, UK

**Keywords:** Psychiatric disorders, Depression, Addiction, Schizophrenia, Bipolar disorder

## Abstract

The successful prevention of mental illness relies upon the identification of causal, modifiable risk factors. However, observational evidence exploring such risk factors often produces contradictory results and randomised control trials are often expensive, time-consuming or unethical to conduct. Mendelian randomisation (MR) is a complementary approach that uses naturally occurring genetic variation to identify possible causal effects between a risk factor and an outcome in a time-efficient and low-cost manner. MR utilises genetic variants as instrumental variables for the risk factor of interest. MR studies are becoming more frequent in the field of psychiatry, warranting a reflection upon both the possibilities and the pitfalls. In this Perspective, we consider several limitations of the MR method that are of particular relevance to psychiatry. We also present new MR methods that have exciting applications to questions of mental illness. While we believe that MR can make an important contribution to the field of psychiatry, we also wish to emphasise the importance of clear causal questions, thorough sensitivity analyses, and triangulation with other forms of evidence.

## Why is causal inference important?

Psychiatric illness presents a worldwide public health problem, with one in four people experiencing a mental illness in their lifetime [1]. In order to reduce this global burden of mental illness, prevention is key. Currently, robust prevention strategies are limited and therefore, we need to identify new modifiable risk factors that can become successful interventions. A pre-requisite for an effective intervention is a causal relationship between the modified risk factor and the risk of mental illness [2]. Although it does not guarantee intervention success, establishing causality is an important first step.

## Why is it difficult to draw causal inference?

Numerous studies have explored potential risk factors for mental illness, but there is consensus for relatively few [3]. In part, this is due to a lack of specific knowledge about the biological pathways underlying mental illness, compared to many physical health conditions (for example, Type 2 diabetes). Without such knowledge, causal inference can be limited, especially if evidence is contradictory. Findings from traditional observational epidemiological studies might be contradictory due to high heterogeneity in mental health presentations, or due to bias from reverse causation and residual confounding. To illustrate this point, let’s take the example of cigarette smoking and depression. Smoking prevalence is much higher amongst individuals with depression than the general population. This could be due to a true causal effect (smoking is a causal risk factor for depression), and/or reverse causation (depression causes individuals to smoke more) and/or confounding (smoking and depression share common risk factors).

The gold standard approach to determine causality would be to conduct a randomised control trial (RCT). However, for certain risk factors (e.g. smoking), it would be highly unethical to randomise individuals to undertake a behaviour we know has adverse physical health consequences. While RCTs for other potential risk factors (e.g. physical activity) might not be unethical, they remain (as with all RCTs) time-consuming and expensive to conduct.

## What is Mendelian randomisation (MR), and can it fix this?

MR is an increasingly popular method, made possible by the wide-spread availability of genotype data. MR can help to identify possible causal risk factors worth prioritising for follow up in RCTs and intervention trials. MR uses genetic variants as proxies for levels of the exposure in an instrumental variable analysis. For example, through genome-wide association studies (GWASs), we might identify genetic variants that pre-dispose individuals to smoke more or fewer cigarettes. These genetic variants can be used as an instrument to test causal effects of the exposure (e.g. smoking) on an outcome (e.g. depression), given that certain assumptions are satisfied (discussed below) [4]. Genetic variants that alter our average lifetime levels of the exposure are randomised at conception and inherited independently of confounding lifestyle factors. This is akin to a natural experiment, and the genetic variants are less likely to be biased by confounding and reverse causation than observed exposures [5]. Therefore, MR analyses can provide causal estimates, as long as the underlying assumptions are satisfied [4].

## Notes of caution when using MR for psychiatry

Although MR has many benefits (i.e. high speed and low cost if suitable genotype and phenotype data are available), there are assumptions and limitations to consider. The three core assumptions of MR are: (1) the relevance assumption—the genetic instrument must be robustly associated with the exposure, (2) the independence assumption—the genetic instrument must not be associated with confounders of the exposure–outcome relationship and (3) the exclusion-restriction assumption—the genetic instrument must only be associated with the outcome via the exposure. These assumptions must hold if we are to make valid causal inference. More detailed discussion of the assumptions is available elsewhere [6, 7].

Here, we focus on limitations and assumption violations that are of particular importance when testing the effect of risk factors on psychiatric phenotypes. The limits of MR for psychiatric outcomes are largely related to the risk factor you wish to investigate. Risk factors with genetic instruments of known biological function pose fewer problems than those with more complex genetic aetiologies. Therefore, to fully explore the limits of MR for psychiatry, we predominantly focus on these latter complex risk factors (which are often behavioural or lifestyle factors).

First, the most important limitation to consider when using psychiatric and behavioural phenotypes is pleiotropy. Pleiotropy occurs when one genetic variant has effects on multiple traits. If these pleiotropic variants affect the outcome through pathways other than via the exposure, then two of the core MR assumptions (independence and exclusion-restriction) could be violated [6, 7]. This will bias estimates by reintroducing confounding. The genetic architecture of psychiatric phenotypes and behavioural risk factors are highly polygenic and pleiotropic [8], and the biological function of their associated genetic variants is often unknown. Consequently, instead of being able to rule out pleiotropic pathways through functional biology, sensitivity analyses must be conducted that are more robust to pleiotropy (discussed in detail elsewhere [9]). The complexity of these phenotypes, and their genetic instruments, means we must be cautious in our interpretation of the results and rigorous in our sensitivity analyses.

Second, the relationships between mental illnesses and behavioural risk factors are plausibly bi-directional. For example, lack of sleep might cause poor mental health and simultaneously, poor mental health might prevent efficient sleep. Bi-directional causal effects present a vicious cycle, which might begin before the onset of diagnosable psychiatric illness. Therefore, understanding bi-directional effects between two traits is important for effective prevention, and should be formally tested using MR where possible. Unfortunately, the interpretation of bi-directional MR is not always straight forward [10]. For example, evidence of a bi-directional relationship could arise because: (1) bi-directional associations are truly causal, (2) genetic instruments for both traits capture an underlying shared risk factor, (3) shared genetic variants act independently on both traits (horizontal pleiotropy) or (4) there is confounding through linkage disequilibrium (LD) [11]. We caution that without understanding the biological function of genetic variants on the two traits of interest, we cannot conclude the presence or direction of a true causal effect. However, approaches including pleiotropy-robust sensitivity analyses [12], multivariable MR (MVMR) [13], colocalization analyses [14], Steiger filtering [15], inspection of LD plots and recently developed methods such as Latent Heritable Confounder MR [16] can help towards ruling out the alternative explanations.

Third, psychiatric phenotypes are highly heterogeneous. For example, depression is a common psychiatric illness, with varied presentations, severity, symptom course and risk factors [17]. Heterogeneity can be a problem for both the exposure and the outcome in MR. As the exposure, genetic instruments for behavioural risk factors (such as smoking) might exert their influence through biological pathways (e.g. neurochemical response to nicotine) or, indirectly, through behavioural pathways (e.g. personality traits of impulsivity and risk taking). Identifying these different pathways could help to better inform intervention targets. Recent methods aimed at identifying sub-groups of instruments that may act though different causal mechanisms, such as the contamination mixture method [18], may prove to be useful when investigating such multifactorial phenotypes. However, we often do not know through which pathways our genetic instruments act and consequently, heterogeneity can be difficult to distinguish from pleiotropy. One way to distinguish the two is the MR Egger intercept test, which can help to quantify the extent the instruments affect the outcome through pleiotropic pathways other than through the exposure [12]. Heterogeneity in the outcome can be a problem because heterogeneous patient groups might require different intervention strategies. Heterogeneity in the outcome will also reduce precision of our causal estimates, making it harder to identify a true causal effect should one exist. Within the field of psychiatric genetics, attention is turning towards the importance of reducing heterogeneity in phenotypes for GWASs [19, 20], and focusing on more homogenous sub-groups will become increasingly possible as sample sizes grow. However, even if more homogenous GWAS were to become a reality, we must not forget that MR is just the first of many steps in developing successful prevention strategies.

Fourth, due to this heterogeneity and the complexity of psychiatric phenotypes, each risk factor alone might explain only a small amount of variance in risk. Consequently, exploring possible modifiable risk factors using MR requires large sample sizes to reach adequate power. When using underpowered GWASs to identify instruments, the criteria for selecting genetic variants are often relaxed, for example including genetic variants associated at *p* < 5 × 10^–7^ rather than at genome-wide significance [21]. Inclusion of variants that are not as robustly associated with the exposure can have the unintended consequence of introducing more pleiotropic pathways and weak instrument bias, while on the other hand, inclusion of more variants is beneficial for several of the more pleiotropy-robust methods and prevents undue weight being placed on individual SNPs that could in fact be pleiotropic. Further MR sensitivity methods can be employed to adjust for possible weak instrument bias, such as Robust Adjusted Profile Scores [22] or methods that do not require *p* value thresholds to select SNPs for inclusion but use genome-wide summary data and statistically account for horizontal pleiotropy (e.g. Causal Analysis Using Summary Effect estimates [23]). Nevertheless, when genetic instruments are only weakly associated with the exposure, we must be cautious in our interpretation of null MR results—an absence of evidence is not evidence of absence—and subsequent MR studies with stronger instruments might reach contradictory findings. Furthermore, although well-powered GWASs are becoming more available, obtaining the required sample size has sometimes come at the cost of detailed measurement of psychiatric illness, and increased phenotype heterogeneity [19]. This has been shown to have a detrimental impact on the specificity of the genetic variants identified [19].

Finally, MR gives an overall causal estimate for a chosen risk factor on a psychiatric illness. Consequently, inferences are limited in their temporality, linearity, generalisability and specificity. Temporality is limited because MR gives estimates of lifetime risk, and hence is not sensitive to critical windows or acute reactions. Linearity is limited because conventional MR estimates linear effects, so might not work for risk factors where we expect a non-linear relationship (e.g. hours of sleep). Extensions of the MR method to account for non-linear effects must be used to detect such relationships [24]. Generalisability is limited because the samples used are often highly selected with the most at risk individuals being the least likely to take part [25]. An assumption of the popular two-sample MR approach is that the exposure and outcome samples are relatively homogenous [26], which can be problematic, when our mental illness data are taken from clinical patient samples and our risk factor data from selected cohort samples. Furthermore, the majority of genetic studies restrict to individuals of European Ancestry, further limiting generalisability of findings [27]. Last, specificity is limited due to the phenotype definitions used in GWASs. GWASs typically combine data from multiple studies and it is often not possible to be overly specific with the phenotype definition, limiting the inferences for interventions. For example, there are genetic variants associated with overall physical activity, but not genetic variants specific to strengthening activity versus cardiovascular activity. These more nuanced questions might be better answered through intervention trials subsequent to MR analyses or perhaps, better powered GWAS samples with deeper phenotyping which will allow us to extend MR to answer these more specific questions in the future.

## Future applications

Whilst keeping in mind these limitations, methods development for MR continues to extend its potential applications. Here, we focus on three recent MR methods that could be applied to important research questions around psychiatry. First, as discussed, mental illness tends to have more diverse risk factors than physical health conditions [17]. These risk factors often do not work in isolation but influence and interact with one another. Understanding the complexity of these causal pathways is important for designing effective interventions. MVMR estimates the direct effect of one risk factor independent of another [13]. For example, this method can be used to separate the effects of education from cognition, and therefore guide intervention targets. As well as identifying independent direct effects, MVMR can also inform us about possible mediation. For example, perhaps smoking influences mental health in part because it influences inflammatory processes. Finally, MVMR can also be used to test possible confounding variables when pleiotropic pathways are suspected [28], as mentioned previously.

Second, when deciding how to intervene, it is informative to know whether it is best to target one risk factor in isolation, or to take a holistic approach and target many. The method of factorial MR can answer this question by estimating cumulative and interaction effects [29]. For example, we could test whether a healthy living intervention targeting smoking, alcohol consumption and physical activity would bring benefits above and beyond targeting each factor individually.

Finally, we are excited about the prospect of applying progression MR to the understanding of psychiatric illness. So far, the MR methods discussed here aim to identify predictors of mental illness onset. While this is crucial to prevent new diagnoses, it does little to help individuals already suffering. There is no reason to assume that the risk factors which cause disease onset will also cause disease progression. For example, smoking is a causal risk factor for lung cancer, but once diagnosed with lung cancer, smoking cessation is not an effective treatment. Standard MR methods are not suitable for addressing questions of progression because they can be biased by selecting on disease incidence [30]. Emerging methods allow the extension of MR to specifically focus on individuals with an existing psychiatric diagnosis, by adjusting for this index event bias [31, 32]. Such extensions could enable us to identify causal risk factors for relapse or factors that predict recovery amongst those with diagnosed mental illness.

## Conclusion

We are optimistic about the contribution of MR to the prevention of mental illness. As more novel genetic instruments become available, we hope that MR can be used to better understand the mechanistic steps along the causal pathway. For example, genetic instruments for emotion recognition processes or serotonergic pathways could move us beyond health behaviours as risk factors, and lead to novel therapeutic targets. However, we hope that this Perspective has emphasised that there are limitations of the MR method and all analyses should be carefully considered and cautiously interpreted. MR studies are easy to conduct, but they are not easy to conduct well. Therefore, we urge for careful study planning, generating a clear hypothesis prior to analysis and conducting rigorous sensitivity analyses. We emphasise that MR is far from a silver bullet. The strongest evidence of a causal effect occurs when multiple methods (each with their own limitations) all reach the same conclusions [33]. Furthermore, MR findings will always require following up in intervention trials to ensure that they ultimately produce successful prevention strategies.
